# Methodological Considerations on the systematic review of dementia care during COVID-19

**DOI:** 10.1590/1980-5764-DN-2025-0309

**Published:** 2025-08-04

**Authors:** Karlos Daniell Araújo dos Santos

**Affiliations:** 1 Universidade Federal de Roraima, Boa Vista RR, Brazil.

Dear Editor,

I would like to present some ­methodological considerations regarding the article “Caring for people with dementia during COVID-19: a systematic review”^1^ recently published in Dementia & Neuropsychologia. While the study provides a relevant overview of the impact of the pandemic on dementia care, we have identified some methodological gaps that may compromise the robustness of the conclusions presented. First, the review was conducted by only two authors, which may limit the diversity of perspectives in study selection and ­evaluation. The presence of a small number of reviewers increases the risk of bias in ­screening and data extraction, as critical decisions may have been made ­without an ­adequate double-checking process^2^ ^,^ ^3^. ­Moreover, the article mentions the assessment of the risk of bias in the included studies but does not ­specify which tool was used for this ­evaluation. Guidelines such as the Cochrane Risk of Bias Tool for ­randomized ­studies^4^ or ROBINS-I for observational studies^5^ are essential for ensuring a ­rigorous assessment of the quality of the studies included. The absence of a detailed analysis compromises the reliability of the findings.

Another relevant methodological aspect is the absence of specific software for review systematization. Tools such as Rayyan, Covidence, or RevMan are widely used to ensure transparency in study selection and data extraction^6^. Without this technological support, there is a greater risk of errors and selection bias. The inclusion criteria of the review also deserve consideration. The study encompassed both qualitative and quantitative methodologies, but it is unclear how the different study types were weighted in the analysis. This lack of ­methodological clarity may affect the interpretation of results, particularly when studies with distinct approaches and varying levels of evidence are considered. Additionally, the review presents a narrative synthesis of the findings without conducting a meta-analysis. While systematic reviews can be informative without a quantitative analysis, the absence of a meta-analysis prevents an estimation of the overall effect of interventions or studied impacts^7^. The inclusion of descriptive statistics or heterogeneity measures would have strengthened the presented findings.

Another critical point is the potential selection bias resulting from the search strategy. The study used five databases (Web of ­Science, Scopus, EBSCOHost, Cochrane Library, and ProQuest) but did not include Medical Literature Analysis and Retrieval System Online/ United States National Library of Medicine (MEDLINE/PubMed), one of the most relevant databases in the biomedical field. The absence of this database may have led to the exclusion of fundamental studies on the topic^8^. It is also important to note that the search was conducted until February 2022. Considering that the COVID-19 pandemic had an ongoing impact and new ­evidence emerged subsequently, the review may not reflect the entirety of the most recent literature on the topic.

Finally, the conclusions of the article could have been more cautious. Although the findings are ­relevant, there is excessive generalization without an in-depth discussion of the limitations of the included ­studies. Low-quality methodological studies may have ­influenced the results, which should have been explicitly highlighted. Given these observations, we suggest that future systematic reviews on the topic incorporate a larger number of reviewers, conduct a structured risk of bias assessment, use systematization software, expand search strategies, and consider conducting a meta-­analysis when possible. These methodological improvements would strengthen the validity of the findings and contribute to a more precise understanding of the challenges faced in dementia care during the pandemic.

We appreciate the opportunity to contribute to the scientific debate and commend the authors for their effort in addressing such a relevant topic.

## REPLY

The authors of the article have been contacted regarding the submission of their reply, but they have not responded to the request from the editorial team.
